# Different Pathological Complete Response Rates According to PAM50 Subtype in HER2+ Breast Cancer Patients Treated With Neoadjuvant Pertuzumab/Trastuzumab vs. Trastuzumab Plus Standard Chemotherapy: An Analysis of Real-World Data

**DOI:** 10.3389/fonc.2019.01178

**Published:** 2019-11-05

**Authors:** Tamara Díaz-Redondo, Rocio Lavado-Valenzuela, Begoña Jimenez, Tomas Pascual, Fernando Gálvez, Alejandro Falcón, Maria del Carmen Alamo, Cristina Morales, Marta Amerigo, Javier Pascual, Alfonso Sanchez-Muñoz, Macarena González-Guerrero, Luis Vicioso, Aurora Laborda, Maria Victoria Ortega, Lidia Perez, Aranzazu Fernandez-Martinez, Nuria Chic, Jose Manuel Jerez, Martina Alvarez, Aleix Prat, Nuria Ribelles, Emilio Alba

**Affiliations:** ^1^Instituto de Investigación Biomédica de Málaga (IBIMA), Hospitales Universitarios Regional y Virgen de la Victoria, Málaga, Spain; ^2^Unidad de Gestión Clínica Oncología Intercentros, Hospitales Universitarios Regional y Virgen de la Victoria, Málaga, Spain; ^3^Laboratorio de Biología Molecular del Cáncer, Centro de Investigaciones Médico-Sanitarias (CIMES), Universidad de Málaga, Málaga, Spain; ^4^Translational Genomics and Targeted Therapeutics in Solid Tumors Lab (IDIBAPS), Hospital Clinic de Barcelona, Barcelona, Spain; ^5^Hospital Universitario Ciudad de Jaén, Jaén, Spain; ^6^Hospital Universitario Virgen del Rocío, Sevilla, Spain; ^7^Hospital Universitario Virgen Macarena, Sevilla, Spain; ^8^Hospital Reina Sofía, Córdoba, Spain; ^9^Hospital Juan Ramón Jiménez, Huelva, Spain; ^10^Hospital Costa del Sol, Marbella, Spain; ^11^Hospital Universitario Puerta del Mar, Cádiz, Spain; ^12^Unidad de Gestión Clínica Anatomía Patológica Intercentros, Hospitales Universitarios Regional y Virgen de la Victoria, Málaga, Spain; ^13^Lineberger Comprehensive Cancer Center, University of North Caroina at Chapel Hill, Chapel Hill, NC, United States; ^14^Departamento de Lenguajes y Ciencias de la Computación, Universidad de Málaga, Málaga, Spain

**Keywords:** breast cancer, real-world data, neoadjuvant, pertuzumab, trastuzumab

## Abstract

**Background:** Double blockade with pertuzumab and trastuzumab combined with chemotherapy is the standard neoadjuvant treatment for HER2-positive early breast cancer. Data derived from clinical trials indicates that the response rates differ among intrinsic subtypes of breast cancer. The aim of this study is to determine if these results are valid in real-world patients.

**Methods:** A total of 259 patients treated in eight Spanish hospitals were included and divided into two cohorts: Cohort A (132 patients) received trastuzumab plus standard neoadjuvant chemotherapy (NAC), and Cohort B received pertuzumab and trastuzumab plus NAC (122 patients). Pathological complete response (pCR) was defined as the complete disappearance of invasive tumor cells. Assignment of the intrinsic subtype was realized using the research-based PAM50 signature.

**Results:** There were more HER2-enriched tumors in Cohort A (70 vs. 56%) and more basal-like tumors in Cohort B (12 vs. 2%), with similar luminal cases in both cohorts (luminal A 12 vs. 14%; luminal B 14 vs. 18%). The overall pCR rate was 39% in Cohort A and 61% in Cohort B. Better pCR rates with pertuzumab plus trastuzumab than with trastuzumab alone were also observed in all intrinsic subtypes (luminal PAM50 41 vs. 11.4% and HER2-enriched subtype 73.5 vs. 50%) but not in basal-like tumors (53.3 vs. 50%). In multivariate analysis the only significant variables related to pCR in both luminal PAM50 and HER2-enriched subtypes were treatment with pertuzumab plus trastuzumab (Cohort B) and histological grade 3.

**Conclusions:** With data obtained from patients treated in clinical practice, it has been possible to verify that the addition of pertuzumab to trastuzumab and neoadjuvant chemotherapy substantially increases the rate of pCR, especially in the HER2-enriched subtype but also in luminal subtypes, with no apparent benefit in basal-like tumors.

## Introduction

The contribution of anti-HER2 therapies to the management of HER2-positive breast cancer patients is undeniable both in metastatic and adjuvant settings (1–6). In the same way, significant benefit was observed with neoadjuvant trastuzumab treatment, obtaining pathological complete response (pCR) rates from 25 to 46% (7–12). These results were improved with the use of pertuzumab combined with trastuzumab, reaching pCR rates between 49 and 69% (8, 13–17).

Several authors have shown that all four intrinsic subtypes can be found in clinically HER2+ tumors. Although the majority of cases are HER2 enriched (40–72%), luminal A (10–27%), luminal B (10–28%), and basal-like tumors (7–14%) are also represented (13, 17–24). This distribution may vary depending on the hormone receptor status. In the hormone-receptor-negative subset, the main intrinsic subtype was HER2 enriched (51–85%), with fewer cases of luminal (luminal A 0.7–24%; luminal B 3–11%), and basal-like subtypes (9–28%) (12, 19, 20, 22, 24, 25). In contrast, in hormone-receptor-positive tumors, luminal subtypes were more frequent (luminal A 28–44%; luminal B 24–48%) than HER2-enriched (8–32%) or basal-like (0.5–2.5%) ones (12, 20, 22, 25, 26).

This heterogeneity is also reflected in the magnitude of the benefit of neoadjuvant anti-HER2 therapies. In the NOAH trial, pCR rate obtained in the trastuzumab arm was higher in the HER2-enriched subtype and in tumors with high ROR scores (18). This association was also observed in patients treated with double HER2 blockade (i.e., trastuzumab plus lapatinib or trastuzumab plus pertuzumab) plus chemotherapy. Neoadjuvant trastuzumab with or without lapatinib shows pCR rates of 50–70% in HER2-enriched tumors, 9–34% in luminal A, 17–36% in luminal B, and 25–38% in basal-like cases (12, 25, 27). Similarly, pCR rates by intrinsic subtype in patients treated with neoadjuvant pertuzumab plus trastuzumab were 70–83% in the HER2-enriched subtype, 16–45% in luminal A, 16–52% in luminal B, and 20–85% in basal-like tumors (13, 17, 22, 23). In a series of patients with BluePrint-defined subtypes, the pCR rate was 76% in the HER2+ type, 31% in the luminal type, and 43% in the basal type (28).

The aim of this work was to evaluate whether the effect of neoadjuvant pertuzumab combined with trastuzumab in comparison with trastuzumab alone varies as a function of PAM50-defined intrinsic subtypes in a real-world cohort of patients with HER2-positive early breast cancer.

## Materials and Methods

### Patients

A total of 254 patients with HER2+ early breast cancer consecutively treated with standard neoadjuvant chemotherapy (NAC) in eight Spanish hospitals were included in the study. The whole population was divided in two cohorts: Cohort A received trastuzumab plus NAC, and Cohort B was treated with pertuzumab and trastuzumab plus NAC. Standard NAC included taxanes with or without anthracyclines. Adjuvant radiotherapy was performed according local practice. Adjuvant endocrine therapy was administered in all hormone-receptor-positive patients.

Patient data were derived from the patients' clinical records and original pathology reports. Although the analysis was retrospective, the data were collected prospectively.

The study was approved by local ethics committees. Written informed consent was obtained from each participant.

### Definition of pCR, Hormone Receptor, HER2 Status, Immunohistochemical Phenotype, and Intrinsic Subtype

pCR was defined as the complete disappearance of invasive tumor cells (ypT0 or ypTis and ypN0). All pathological determinations were performed on diagnostic biopsies. Tumors were classified as estrogen-receptor and progesterone-receptor-positive if ≥1% of tumor cells were stained. HER2+ status was defined by an immunohistochemistry score of 3+ or a HER2 amplification ratio of 2.0 or more by FISH or SISH. Hormone-receptor-positive cases were classified as Luminal-HER2 (luminal immunophenotype) and those with negative hormone receptors such ones as HER2+ (HER2+ immunophenotype).

Assignment of the intrinsic subtype was realized using the research-based PAM50 signature as previously described (29) in order to categorize all cases as one of the following subtypes: luminal A, luminal B, HER2-enriched, basal-like, and normal-like.

### Statistical Analysis

Associations between variables and pCR were evaluated by the chi-squared test or Fisher's exact test. Multivariate logistic regression analyses were used to evaluate the association of each intrinsic subtype with pCR and included the variables that showed significant associations in univariate analyses. Cases with unknown data for any of the variables considered were excluded from multivariate analyses.

All the tests were two-sided, and a *P*-value of < 0.05 was considered to indicate statistical significance. Analyses were carried out using the R system for statistical computing (version 3.5.2).

## Results

### Patients' Characteristics

A total of 254 patients were included in the study: 132 patients were treated with NAC plus trastuzumab (Cohort A) and 122 patients with NAC plus pertuzumab and trastuzumab (Cohort B). The clinical characteristics are outlined in Table 1. There were more cases with a greater tumor burden in Cohort B than in Cohort A (T3 or T4 tumor size: 33 vs. 19%; stage I: 1 vs. 14%). Slightly more patients were treated with taxanes alone in Cohort B (14 vs. 7%).

**Table 1:** Patient characteristics.

	**Cohort A (T)**		**Cohort B (P+T)**	
**Total**	132		122	
**Menopausal status**				
Premenopausal	57	43%	57	47%
Postmenopausal	50	38%	52	43%
NA	25	19%	13	11%
**Age**				
<50	63	48%	58	48%
≥50	69	52%	64	52%
**Tumor size**				
T1	26	20%	14	11%
T2	78	59%	67	55%
T3	15	11%	27	22%
T4	10	8%	13	11%
NA	3	2%	1	1%
**Nodal status**				
Negative	55	42%	44	36%
Positive	77	58%	77	63%
NA			1	1%
**Stage**				
I	18	14%	1	1%
II	90	68%	90	74%
III	21	16%	30	25%
NA	3	2%	1	1%
**Grade**				
1–2	62	47%	37	30%
3	45	34%	45	37%
ND	25	19%	40	33%
**Hormone receptor**				
Negative	41	31%	48	39%
Positive	91	69%	74	61%
**Ki67**				
<20	25	19%	13	11%
20–50	44	33%	76	62%
>50	29	22%	30	25%
ND	34	26%	3	2%
**NAC**				
Taxanes^a^	9	7%	17	14%
Taxanes + Anthracyclines^b^	123	93%	105	86%
**pCR**				
No	80	61%	48	39%
Yes	52	39%	74	61%
**Immunohistochemical phenotype**				
Luminal-HER2	91	69%	74	61%
HER2	41	31%	48	39%
**PAM50-based subtype**				
Luminal A	16	12%	17	14%
Luminal B	19	14%	22	18%
HER2-enriched	92	70%	68	56%
Basal-like	2	2%	15	12%
Normal	3	2%		

*NA, Not available*.

a *Paclitaxel-Trastuzumab; Docetaxel-Trastuzumab; Paclitaxel-Trastuzumab-Pertuzumab; Docetaxel-Trastuzumab-Pertuzumab*.

b *Epirrubicin-Cyclophosphamide followed by Docetaxel-Trastuzumab; Epirrubicin-Cyclophosphamide followed by Paclitaxel-Trastuzumab; Adriamycin-Cyclophosphamide followed by Docetaxel-Trastuzumab; Adriamycin-Cyclophosphamide followed by Paclitaxel-Trastuzumab; Fluorouracil-Epirrubicin-Cyclophosphamide followed by Docetaxel-Trastuzumab; Fluorouracil-Epirrubicin-Cyclophosphamide followed by Paclitaxel-Trastuzumab; Epirrubicin-Cyclophosphamide followed by Docetaxel-Trastuzumab-Pertuzumab; Epirrubicin-Cyclophosphamide followed by Paclitaxel-Trastuzumab-Pertuzumab; Adriamycin-Cyclophosphamide followed by Docetaxel-Trastuzumab-Pertuzumab; Adriamycin-Cyclophosphamide followed by Paclitaxel-Trastuzumab-Pertuzumab; Fluorouracil-Epirrubicin-Cyclophosphamide followed by Docetaxel-Trastuzumab-Pertuzumab; Fluorouracil-Epirrubicin-Cyclophosphamide followed by Paclitaxel-Trastuzumab-Pertuzumab*.

The overall pCR rate was 39% in Cohort A and 61% in Cohort B. The immunohistochemical phenotype distribution was similar in both cohorts: luminal-HER2 69 vs. 61% and HER2+ cases 31 vs. 39%. Regarding PAM50-assigned subtypes, there were more HER2-enriched tumors in Cohort A (70 vs. 56%) and more basal-like tumors in Cohort B (12 vs. 2%), with similar luminal case distributions (luminal A 12 vs. 14%; luminal B 14 vs. 18%).

### Association Between Variables and pCR

In the whole population (Table 2), pCR was significantly related to the type of treatment (Cohort A 39.4% vs. Cohort B 60.6%; *P* = 0.0011), histological grade (grade 1 + 2 35.5% vs. grade 3 62.2%; *P* = 0.0007), Ki67 level (<20% 28.9% vs. 20–50% 60.8% vs. >50% 54.2%; *P* = 0.003), immunohistochemical phenotype (luminal HER2 38.7% vs. HER2+ 69.6%; *P* = 0.000005), and PAM50-based subtype (luminal A 21.2% vs. luminal B 31.7% vs. HER-2 enriched 60% vs. basal like 52.9%; *P* = 0.0004). Similar results were observed in separate analyses of each cohort (Table 2).

**Table 2:** Association between variables and pCR.

	**Whole population**		**Cohort A (T)**		**Cohort B (P+T)**	
	**pCR (%)**	***P***	**pCR (%)**	***P***	**pCR (%)**	***P***
Cohort A	39.4	0.001				
Cohort B	60.6					
Grade 1–2	35.5	0.00007	27.4	0.038	40.5	0.002
Grade 3	62.2		48.9		75.5	
Ki67 <20%	28.9	0.002	20.0	0.006	46.1	0.4
Ki67 20–50%	60.8		56.8		63.1	
Ki67 >50%	54.2		41.3		66.0	
Luminal-HER2	38.7	0.000005	30.7	0.004	48.6	0.001
HER2	69.6		58.5		79.1	
Luminal A	21.2	0.00004	6.2	0.0004	35.2	0.007
Luminal B	31.7		15.7		45.4	
HER2-E	60.0		50.0		73.5	
Basal-like	52.9		50.0		53.3	
Normal	33.3		33.3		0.0	

The better results found in cohort B in the whole population were also observed in an evaluation of different subpopulations (Table 3). Thus, immunohistochemical luminal tumors showed greater pCR with pertuzumab and trastuzumab treatment (48.6 vs. 30.8%; *P* = 0.03) and also HER2+ patients (58.5 vs. 79.6%; *P* = 0.06). In addition, in the luminal PAM50-based subtype, a pCR rate of 11.4% was obtained with trastuzumab treatment vs. 41% with combination treatment (*P* = 0.008) and in the HER2-enriched subtype, these rates were 50 vs. 73.5% (*P* = 0.004).

**Table 3:** Association between variables and pCR in specific subpopulations.

	**pCR (%)**	***P***
**Luminal-HER2 immunophenotype**		
Cohort A	30.8	0.03
Cohort B	48.6	
**HER2+** **immunophenotype**		
Cohort A	58.5	0.06
Cohort B	79.6	
**Luminal PAM50 subtypes**		
Cohort A	11.4	0.008
Cohort B	41.0	
**HER2-enriched PAM50 subtypes**		
Cohort A	50.0	0.004
Cohort B	73.5	

### Multivariate Analyses

The variables that remained significantly associated with pCR in the whole population were treatment Cohort B [Odds Ratio (OR) 2.5; 95% CI 1.07–6; *P* = 0.036], histological grade 3 (OR 3.41; 95% CI 14.48–8.09; *P* = 0.004), immunophenotype HER2+ (OR 3.82; 95% CI 1.39–11.6; *P* = 0.01), and PAM50-based HER2-enriched subtype (OR 2.98; 95% CI 1.39–11.6; *P* = 0.02) (Table 4).

**Table 4:** Multivariate logistic regression of pCR.

	**Whole population**			**Cohort A (T)**			**Cohort B (P+T)**			**Luminal PAM50**			**HER2-E PAM50**		
	**OR**	**95% CI**	***P***	**OR**	**95% CI**	***P***	**OR**	**95% CI**	***P***	**OR**	**95% CI**	***P***	**OR**	**95% CI**	***P***
Cohort B	2.5	1.07–6.00	0.03	–	–	–	–	–	–	4.2	1.05–22.4	0.05	2.7	1.01–7.6	0.05
Grade 3	3.41	1.48–8.09	0.004	5.1	1.5–20.7	0.01	3.4	1.1–10.8	0.03	4.5	1.1–19.0	0.03	4.1	1.6–11.2	0.003
HER2ihc	3.82	1.39–11.6	0.01	9.8	2.0–75.3	0.01	–	–	–	–	–	–	–	–	–
HER2-E	2.98	1.19–7.7	0.02	–	–	–	3.7	1.2–11	0.02	–	–	–	–	–	–

*OR, odds ratio; CI, Confidence Interval; HER2ihc, immunophenotype HER2; HER2E, HER2 enriched*.

In the cohort of patients treated with trastuzumab alone, grade 3 (OR 5.1; 95% CI 1.5–20.7; *P* = 0.01) and immunophenotype HER2+ (OR 9.8; 95% CI 2.0–75.3; *P* = 0.01) were the only variables independently associated with a higher probability of pCR, and in the cohort of patients that received pertuzumab and trastuzumab, these variables were grade 3 (OR 3.4; 95% CI 1.1–10.8; *P* = 0.03) and PAM50-based HER2-enriched subtype (OR 3.7; 95% CI 1.2–11; *P* = 0.02) (Table 4).

In an analysis of luminal PAM50-based tumors, the variables that remained significantly associated with pCR were treatment Cohort B (OR 4.2; 95% CI 1.05–22.4; *P* = 0.05), and grade 3 (OR 4.5; 95% CI 1.1–19.0; *P* = 0.03); this was also true in the HER2-enriched subgroup (Cohort B OR 2.7; 95% CI 1.01–7.6; *P* = 0.05. Grade 3 OR 4.1; 95% CI 1.6–11.2; *P* = 0.003) (Table 4).

## Discussion

Our study provides valuable information from the real world about neoadjuvant anti-HER2 treatment in early breast cancer, showing that the rate of pCR obtained by double blockade with pertuzumab plus trastuzumab exceeds by 20% that obtained with trastuzumab alone. The pCR rate observed in our series with pertuzumab and trastuzumab treatment (60.6%) is in the range of responses observed in the published phase II-III trials (45.8–69.8%) (8, 13–15, 17, 22). Moreover, the pCR rate found in patients treated with trastuzumab alone (39.4%) is in agreement with previous data (31–46%) (7–12). Interestingly, the greater efficacy shown by the combination of pertuzumab and trastuzumab in our study was despite the fact that the patients in this cohort had worse prognostic characteristics than those who received trastuzumab alone, with a higher percentage of tumors larger than 5 cm or a greater number of cases with nodal involvement. Lower pCR rates were observed in patients with the luminal immunophenotype in both the cohort treated with pertuzumab and trastuzumab and in the one receiving trastuzumab alone. This finding is consistent with previously published data (8, 9, 12, 15, 17, 28).

Although most tumors positive for HER2 by immunohistochemistry or by *in situ* hybridization correspond to the intrinsic HER2-enriched subtype, it is possible to identify any of the remaining intrinsic subtypes in this type of tumor (19, 29, 30). Surprisingly, the percentage of cases by intrinsic subtype in our two patient cohorts differ to some extent, despite the fact that the processing of the tumor samples was performed in the same laboratory, albeit at different times. In the group of patients treated with pertuzumab and trastuzumab, 56% of the cases corresponded to the HER2-enriched, 14% to the luminal A, 18% to the luminal B, and 12% to the basal-like subtype, and this distribution is in agreement with previously published data (13, 17, 18, 20, 22, 23). However, in the cohort of patients who received trastuzumab alone, there was a higher percentage of HER2-enriched cases (70%), a lower number of basal-like tumors (2%), and a similar amount of luminal tumors (luminal A 12%; luminal B 14%). Similar data were reported by Perez et al. from NCCTG N9831 Trial (21) and more recently by Tolaney et al. from the APT Trial (24).

Anti-HER2 therapies are more beneficial in HER2-enriched tumors, but all intrinsic subtypes benefit from this type of treatment in both the adjuvant (20, 21) and neoadjuvant settings, and the HER2-enriched subtype benefits the most (13, 17, 18, 22, 23, 28). According to these data, our patients with HER2-enriched tumors obtained the highest pCR rate with both treatment schedules. Furthermore, the use of pertuzumab and trastuzumab was the only variable, together with the histological grade, that provided independent predictive information for pCR events in both HER2-enriched tumors (OR 2.7) and patients with luminal subtypes (OR 4.2). Although the number of patients was small, the basal-like subtype shows no benefit with the use of anti-HER2 therapy, achieving nearly the same pCR rate with pertuzumab and trastuzumab as with trastuzumab alone.

To our knowledge, there is no published series of real-world patients with early HER2+ breast cancer treated with NAC plus pertuzumab and trastuzumab or trastuzumab alone, in which the intrinsic subtypes have been established according to the PAM50 definition and their relationship with the pCR rate analyzed. Beitsch et al. (28) published data from patients included in a prospective registry, of whom 178 were treated with NAC plus trastuzumab and 119 with NAC plus pertuzumab and trastuzumab and in which the molecular subtype was defined by BluePrint platform. Their results agree with ours, showing a higher response with double HER2 blockade vs. treatment with trastuzumab alone in the HER2+ type (76% vs. 57%) and the luminal type (31% vs. 8%) and no differences in the basal type (43% vs. 45%). Recently, Fashing et al. (31) published their results from a series of patients included in an ongoing registry comparing two cohorts of patients that received neoadjuvant treatment with chemotherapy plus trastuzumab or chemotherapy plus trastuzumab and pertuzumab. In agreement with our results, there was a greater number of pCR in patients treated with pertuzumab plus trastuzumab with an adjusted OR for double HER2 blockade vs. trastuzumab alone of 2.04 (95% CI 1.24–3.35).

Our results confirm the data obtained from clinical trials in patients treated in clinical practice, showing that the addition of pertuzumab to trastuzumab and neoadjuvant chemotherapy increase the pCR rate substantially, especially in the HER2-enriched subtype but also in luminal subtypes, with no apparent benefit in basal-like tumors.

## Data Availability Statement

The raw data supporting the conclusions of this manuscript will be made available by the authors, without undue reservation, to any qualified researcher.

## Ethics Statement

The studies involving human participants were reviewed and approved by Comité de Ética de la Investigación Provincial de Málag, Servicio Andaluz de Salud, Consejería de Salud, Junta de Andalucía. The patients/participants provided their written informed consent to participate in this study.

## Author Contributions

EA, TD-R, and AP made substantial contributions to the conception of the work. TD-R, RL-V, BJ, TP, FG, AF, MCA, CM, MAl, JP, AS-M, MG-G, LV, AL, MO, LP, AF-M, NC, and MAm contributed to the acquisition of the data. EA, JJ, TD-R, and NR contributed to the analysis of data. NR and EA drafting the work. All authors approved the final version of the manuscript.

### Conflict of Interest

The authors declare that the research was conducted in the absence of any commercial or financial relationships that could be construed as a potential conflict of interest.
